# Antimicrobial and Anti-Efflux Machinery of FDA-Approved Proton Pump Inhibitors and Vitamins Against *Klebsiella pneumoniae* and *Pseudomonas aeruginosa*

**DOI:** 10.3390/microorganisms13061227

**Published:** 2025-05-27

**Authors:** Lekaa L. Lutfi, Moataz A. Shaldam, Mona I. Shaaban, Soha Lotfy Elshaer

**Affiliations:** 1 Department of Microbiology and Immunology, Faculty of Pharmacy, Mansoura University, Mansoura 35516, Egypt; lekaalutfi1@std.mans.edu.eg (L.L.L.); sohaaelshaer@yahoo.com (S.L.E.); 2 Department of Microbiology and Immunology, Faculty of Pharmacy, Horus University-Egypt, New Damietta 34518, Egypt; 3 Department of Pharmaceutical Chemistry, Faculty of Pharmacy, Kafrelsheikh University, Kafr El Sheikh 33516, Egypt; dr_moutaz_986@pharm.kfs.edu.eg

**Keywords:** *K. pneumoniae*, *P. aeruginosa*, efflux pump, esomeprazole, omeprazole, pantoprazole, vitamin D, vitamin K

## Abstract

Background: The efflux system is one of the resistance mechanisms that bacteria use to reduce the effectiveness of antibiotics, leading to the development of multidrug resistance. To evaluate other treatment choices, esomeprazole (ESO), omeprazole (OME), pantoprazole (PAN), vitamin D (VD), and vitamin K (VK) were tested for potential efflux pump (EP)-inhibiting activity. Methods: The minimum inhibitory concentrations (MICs) of the tested drugs were determined against *K. pneumoniae* ATCC 51503 and *P. aeruginosa* PAO1. Quantitative estimation of the EP-inhibiting activity of the tested medications was phenotypically investigated with a semi-automated fluorometric system and genotypically confirmed by real-time polymerase chain reaction (RT-PCR). Data were confirmed through docking study. Results: *K. pneumoniae* ATCC 51503 and *P. aeruginosa* PAO1 were positive efflux standard strains. VD and VK revealed an MIC_VD_ of 625–1250 µg/mL and MIC_VK_ of 2500–5000 µg/mL, lower than what was detected for PPIs (MIC_PPIs_ = 16,000–32,000 µg/mL). Vitamins showed powerful anti-efflux activity with remarkable ethidium bromide accumulation in *K. pneumoniae* ATCC 51503 and *P. aeruginosa* PAO1. Also, VD and VK significantly lowered the MIC of ciprofloxacin by 64-fold. On the molecular level, OME showed a notable decrease in the relative expression of the efflux-encoding genes *acrB* and *mexA* by 91.5% and 99.7% in ATCC 51503 and PAO1, respectively. Conclusion: This study highlights the anti-efflux activity of ESO, OME, PAN, VD, and VK against the tested Gram-negative strains. Hence, these PPIs and vitamins could be valuable adjuvant treatments to enhance the effectiveness of curing infections caused by MDR strains.

## 1. Introduction

*Enterobacteriaceae*, including *Klebsiella pneumoniae* (*K. pneumoniae*) and *Pseudomonas aeruginosa* (*P. aeruginosa*), are among the prime pathogens on the high-priority pathogenic list published by the World Health Organization (WHO) since 2017 [1,2]. These pathogens pose a high risk due to their ability to spread widely in the host, causing life-threatening opportunistic and nosocomial infections. They are part of the ESKAPE list (*Enterococcus faecium*, *Staphylococcus aureus*, *K. pneumoniae*, *Acinetobacter baumannii*, *P. aeruginosa*, and *Enterobacter* spp.) as they evade the activity of various antibiotics, leading to a global health crisis [3]. These pathogens also rapidly evolve into multidrug-resistant strains, resulting in prolonged hospital stays and increased mortality rates [4].

These pathogens possess diverse resistance mechanisms, either acquired or innate, such as drug-target mutations, enzymatic-based drug inactivation, and a decrease in the intracellular effective drug concentration, either by decreasing drug entry or increasing its efflux to the cell surroundings [5]. The efflux pump is a proteinaceous structure that is considered a cause of multidrug resistance (MDR) on its own. It has a broad substrate profile that can expel multiple antibiotics from similar or different classes due to their structural similarities. Alongside this, it can augment bacterial resistance levels through synergistic interactions with other resistance mechanisms [6], thereby prolonging microbial survival in challenging environmental conditions [7].

Until now, two categories of efflux system have been identified, comprising six major classes. The primary category derives its energy from ATP hydrolysis such as the ATP-binding cassette (ABC). The secondary category acts through proton exchange or ion-dependent gradients and includes five extrusion pumps: resistance–nodulation–cell division (RND), small multidrug resistance (SMR), major facilitator superfamily (MFS), multidrug and toxins extrusion (MATE), and the proteo-bacterial antimicrobial complex efflux family (PACE) [8]. The tripartite RND transporter is the well-recognized efflux type, found only in Gram-negative bacteria, such as AcrAB-TolC in most *Enterobacteriaceae* strains [9,10] and its homologue, the MexAB-OprM pump in *P. aeruginosa* [11], both contributing to resistance to almost every antimicrobial class.

Since the majority of antimicrobial resistance (AMR) genes are located on plasmids and other genetically transferable elements, this makes *K. pneumoniae* and *P. aeruginosa* act as “key sellers” for the development and sharing of these resistant traits with other species [12]. Therefore, targeting the efflux system is a critical therapeutic strategy to combat the spread of AMR in these two notorious pathogens.

Numerous efflux pump inhibitors (EPIs) such as carbonyl cyanide m-chlorophenyl hydrazone (CCCP) [13], berberine [14], quercetin [15], and the synthetic ML-7 compound [16] have been evaluated against MDR *K. pneumoniae*. Also in *P. aeruginosa*, potent EPIs have been tested, including epigallocatechin-3-gallate [17], glycyrrhizin [18], ethyl 4-bromopyrrole-2-carboxylate (RP1) [19], and an indole formamide TXA01182 compound [20], in addition its conformational analogue TXA09155, which enhanced the effectiveness of several antimicrobials in wild-type *P. aeruginosa* and clinical isolates as well [21]. Some of these inhibitors had a limited effect, while others had a remarkable activity upon coupling with antimicrobial agents.

To effectively block bacterial pumping-out mechanisms, EPIs should possess a broad spectrum of activity against multiple pumps, high bioavailability, minimal toxicity, and no induction of AMR [22]. Unfortunately, due to the lack of the aforementioned properties, unstudied pharmacokinetic and pharmacodynamic profiles, the lower activity of EPI when combined with a specific antibiotic, and the time required to discover a suitable new and qualified EPI, no EPI has progressed to clinical research trials [23].

Recently, many Food and Drug Administration (FDA)-approved medications have been screened for possible NorA EPI activities in *S. aureus*, like small molecular compounds that potentiate fluoroquinolone resistance [24] in addition to diclofenac sodium and domperidone [25]. Furthermore, phentolamine, used primarily to treat severe hypertensive states, was discovered to boost the macrolide activity towards MDR Gram-negative-associated infections [26].

Medications that have been approved for marketing by the FDA are known for their notable pharmacological safety and in vivo therapeutic efficacy, making them safe for administration. However, few studies have addressed the influence of proton pump inhibitors on targeting the bacterial efflux in Gram-negative bacteria. Also, the efflux-impeding activity of vitamins D and K3 was studied on Gram-positive *Streptococcus mutans* [27] and *S. aureus* [28], respectively.

Therefore, this research was performed to study some FDA-approved proton pump inhibitors (esomeprazole (ESO), omeprazole (OME) and pantoprazole (PAN)) and vitamins (vitamin D and vitamin K) for efflux pump inhibitory effects in two medically important organisms, *K. pneumoniae* and *P. aeruginosa*.

## 2. Results

### 2.1. PCR Confirmation for Efflux Pump Genes

**Figure 1:**
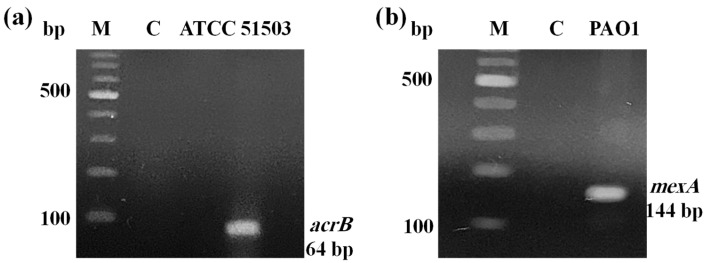
Agarose gel electrophoresis of efflux-encoding genes: (**a**) *acrB* in *K. pneumoniae* ATCC 51503 and (**b**) *mexA* in *P. aeruginosa* PAO1. Lane (M) is the 100 bp DNA ladder and lane (C) is the negative control reaction.

PCR analysis of genes responsible for efflux-encoding activity revealed that *K. pneumoniae* ATCC 51503 harboured its representative gene, *acrB*, at 64 bp (Figure 1a). Also, the *mexA* gene was carried by PAO1 with amplicon sizes of 144 bp (Figure 1b).

### 2.2. The Minimum Inhibitory Concentrations of the Tested Compounds Against the Gram-Negative Standard Strains

The MICs of the three PPIs (ESO, OME, and PAN) and two vitamins (VD and VK), in addition to a reference efflux substrate (EB) and inhibitor (CCCP) against two Gram-negative standard strains of *K. pneumoniae* ATCC 51503 and *P. aeruginosa* PAO1, were determined by microbroth dilution method and are given in Table 1.

The MIC values of EB were 2 μg/mL and 32 μg/mL for *K. pneumoniae* ATCC 51503 and *P. aeruginosa* PAO1, respectively, whereas CCCP MIC was 16,000 μg/mL for both strains. As can be observed in Table 1, the three PPIs exhibited the same MIC value (16,000 µg/mL) against the tested strains, except that ESO and PAN in PAO1 was 32,000 µg/mL.

For vitamins, the MIC of VD was 625 µg/mL for *K. pneumoniae* ATCC 51503 and 1250 µg/mL for PAO1, while the MIC of VK in *K. pneumoniae* ATCC 51503 was 2500 µg/mL, which was less than that assigned to PAO1 (MIC = 5000 µg/mL) by half. Sub-MIC values (0.5× and 0.25× MIC) of PPIs and vitamins were displayed as well (Table 1).

### 2.3. Fluorometric Real-Time Estimation of Efflux Activity

#### 2.3.1. EB Accumulation Assay

*K. pneumoniae* ATCC 51503 and *P. aeruginosa* PAO1 were tested to characterize their efflux properties and detect the EPI activities of the tested drugs.

The initial step in this technique is to establish the ideal conditions for accumulation of EB inside the bacterial cells followed by selecting the appropriate equilibrium EB concentration above which there is detectable accumulation within the cells. Accumulation of different EB concentrations was evaluated in the absence and presence of glucose, and one-way ANOVA test was performed to determine whether there was a statistical difference between both treatment conditions.

**Figure 2:**
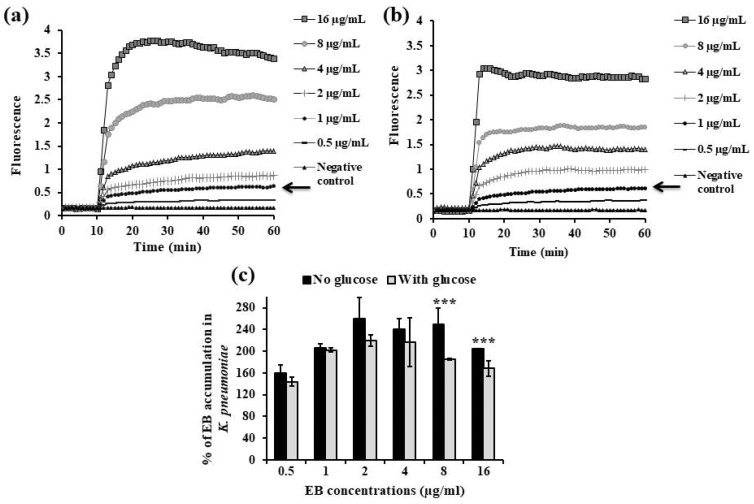
Accumulation of increasing concentrations of ethidium bromide (0.5–16 µg/mL) in *K. pneumoniae* ATCC 51503 in (**a**) the absence of glucose and (**b**) the presence of glucose. The minimum concentration of EB was detected as the concentration that promoted an equilibrium between influx and efflux of EB substrate over 60 min (it is indicated in each graph by an arrow). (**c**) Accumulation percentage of EB in the absence and presence of glucose. The error bars indicate the SD from the mean of duplicate experiments. The asterisks indicate highly significant differences, with *** *p* < 0.0001 between both treatment conditions.

In *K. pneumoniae* ATCC 51503, the maximum concentration of EB that can be extruded by this organism in either the absence (Figure 2a) or presence (Figure 2b) of glucose is 1 µg/mL, since more EB accumulation will occur above this concentration. There were no significant differences in the amounts of EB accumulated by the *K. pneumoniae* ATCC 51503 strain with no glucose and cells exposed to glucose throughout the EB concentration range except for 8 and 16 µg/mL, as the % accumulation was 250% vs. 185% and 204% vs. 168%, respectively (*p* ˂ 0.0001, Figure 2c).

**Figure 3:**
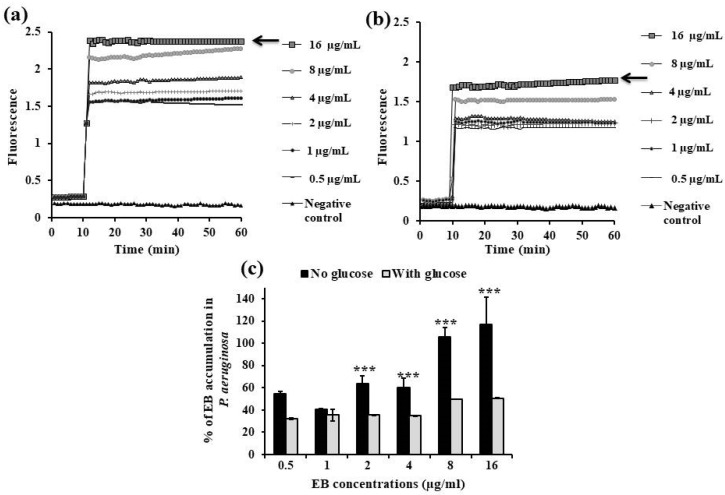
Accumulation of increasing concentrations of ethidium bromide (0.5–16 µg/mL) in *P. aeruginosa* PAO1 in (**a**) the absence of glucose and (**b**) the presence of glucose. The minimum concentration of EB was detected as the concentration that promoted an equilibrium between influx and efflux of EB substrate over 60 min (it is indicated in each graph by an arrow). (**c**) Accumulation percentage of EB in the absence and presence of glucose. The error bars indicate the SD from the mean of duplicate experiments. The asterisks indicate highly significant differences, with *** *p* < 0.0001 between both treatment conditions.

As shown in Figure 3a,b, the lowest concentration that resulted in equilibrium between the influx and efflux of EB substrate in *P. aeruginosa* PAO1, in the absence or presence of glucose, was 16 µg/mL. When compared with glucose-treated PAO1, the accumulation percentage of EB at 2 µg/mL (35%), 4 µg/mL (35%), 8 µg/mL (50%), and 16 µg/mL (51%) significantly increased by 64%, 60%, 106%, and 117%, respectively, in cells without glucose (*p* ˂ 0.0001, Figure 3c).

#### 2.3.2. Evaluation of EPI Activities of the Tested PPIs and Vitamins

The ability of the two reference strains to efflux EB was assessed in the presence of FDA-approved PPIs and vitamins using a known EPI (CCCP) as a proof of concept, and the EB efflux was represented in terms of relative final fluorescence (RFF). Each strain was tested, in triplicate, at its equilibrium EB concentration and in the absence and presence of 0.5× MIC of each drug (Table 1). All assays were performed in the presence of 0.4% glucose.

**Figure 4:**
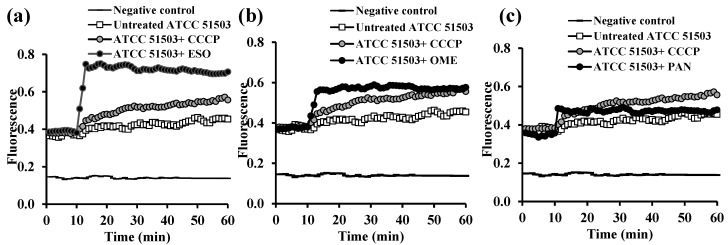
Assessment of ethidium bromide accumulation at its equilibrium concentration in the presence and absence of 0.5× MIC of proton pump inhibitors (**a**) esomeprazole (ESO), (**b**) omeprazole (OME), and (**c**) pantoprazole (PAN) in *K. pneumoniae* ATCC 51503 standard strain compared with the effect of carbonyl cyanide m-chlorophenyl hydrazone (CCCP).

The accumulation of EB (1 µg/mL) in *K. pneumoniae* ATCC 51503 cells after the addition of either CCCP or any PPI agent within 10 min (ESO (Figure 4a), OME (Figure 4b), and PAN (Figure 4c)) was significantly higher (*p* < 0.0001) than in the untreated strain. Unlike PAN, ESO and OME displayed RFF values of 0.58 ± 0.06 and 0.29 ± 0.13, respectively, against the *K. pneumoniae* ATCC 51503 strain, indicating their ability for higher levels of EB accumulation than CCCP (RFF = 0.18 ± 0.09, Table 2).

**Figure 5:**
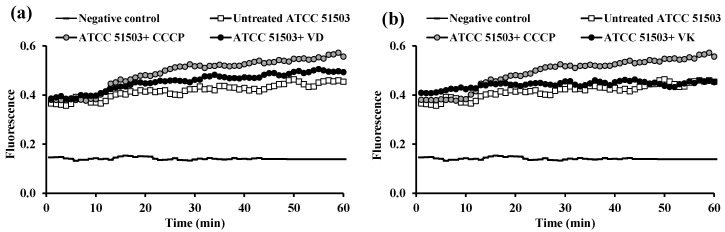
Assessment of ethidium bromide accumulation, at its equilibrium concentration, in the presence and absence of 0.5× MIC of vitamins (**a**) vitamin D (VD) and (**b**) vitamin K (VK) in *K. pneumoniae* ATCC 51503 standard strain compared with the effect of carbonyl cyanide m-chlorophenyl hydrazone (CCCP).

The fluorescence value of *K. pneumoniae* ATCC 51503 cells in the presence of VD (Figure 5a) and VK (Figure 5b) was slightly more than the control strain; however, the efflux activity of both vitamins was less (lower RFF values) than that of CCCP (RFF was 0.092 ± 0.04 for VD and 0.06 ± 0.04 for VK versus 0.18 ± 0.09 for CCCP, *p* < 0.0001) (Table 2).

**Figure 6:**
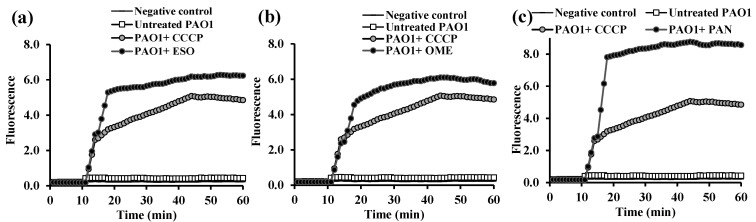
Ethidium bromide accumulation assay in *P. aeruginosa* PAO1 treated with proton pump inhibitors, (**a**) esomeprazole (ESO), (**b**) omeprazole (OME), and (**c**) pantoprazole (PAN), used at 0.5× of their MICs, compared with PAO1-treated carbonyl cyanide m-chlorophenyl hydrazone (CCCP) and untreated PAO1 strain.

All three PPIs, at the sub-inhibitory concentration, demonstrated a substantial increase in EB accumulation by *P. aeruginosa* PAO1 (16 µg/mL) (Figure 6), even higher than the positive control, CCCP. The fold change in EB accumulation of ESO was slightly higher than that of CCCP (RFF = 21.03 ± 2.2 vs. 15.6 ± 1.8) and OME (RFF = 19.7 ± 1.3), and it was significantly higher (2-fold increase) for PAN (RFF = 29.9 ± 2.8, *p* = 0.001) (Table 3).

**Figure 7:**
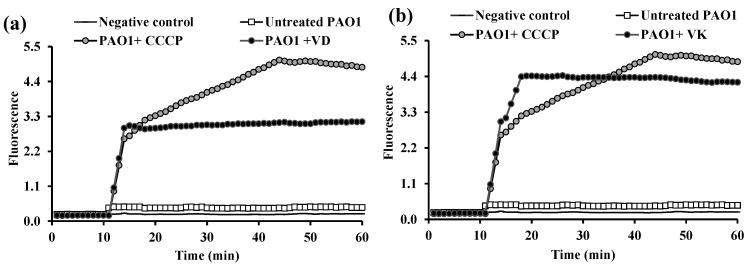
Ethidium bromide accumulation assay in *P. aeruginosa* PAO1 treated with vitamins, (**a**) vitamin D (VD) and (**b**) vitamin K (VK), used at 0.5× of their MICs, compared with PAO1-treated carbonyl cyanide m-chlorophenyl hydrazone (CCCP) and untreated PAO1 strain.

Similarly, challenged PAO1 (after 10 min incubation) with either vitamin, VD (Figure 7a) or VK (Figure 7b), resulted in significant increase in EB detainment compared with the control PAO1, *p* < 0.0001. Compared with the RFF of CCCP (15.6 ± 1.8), a very similar RFF value for VK (15.1 ± 1.3), but a low RFF value for VD (10.8 ± 1.4), were shown (Table 3).

### 2.4. The MIC of Ciprofloxacin Alone and in Combination with the Tested Compounds

The MIC of ciprofloxacin against both *K. pneumoniae* ATCC 51503 and *P. aeruginosa* PAO1 was 62.5 µg/mL (Table 4). The presence of 0.5× MICs of either VD or VK with ciprofloxacin significantly reduced the MIC of ciprofloxacin in both *P. aeruginosa* PAO1 to 0.98–1.95 µg/mL and *K. pneumonia* ATCC 51503 to 3.9–7.8 µg/mL (Table 4).

Furthermore, the combination of ciprofloxacin with PPEs showed a significant decrease in the MIC of ciprofloxacin, to 0.98 µg/mL, with *P. aeruginosa* PAO1 *isolate* and to 0.98–15.6 µg/mL ciprofloxacin/PPE combinations with *K. pneumonia* ATCC 51503 (Table 4).

### 2.5. RT-PCR Assessment for Efflux Gene Quantification

The expression level of *acrB* and *mexA* efflux genes in challenged *K. pneumoniae* ATCC 51503 and *P. aeruginosa* PAO1 with 0.5× MICs of each tested PPI, respectively, was quantified and further normalized relative to the expression levels of the standard specialized housekeeping genes, *ropD* in the same strain, in accordance with the 2^−∆∆CT^ analysis method.

**Figure 8:**
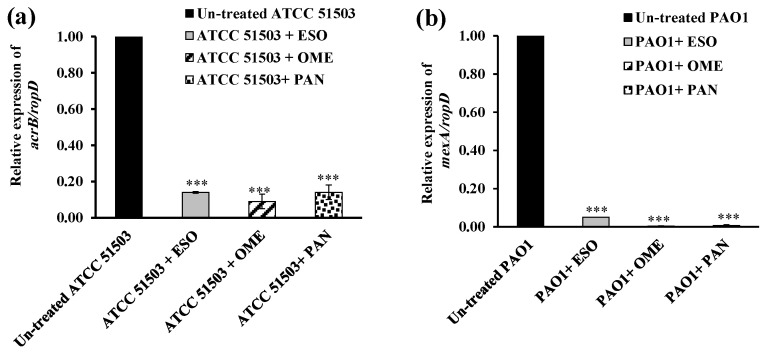
Relative expression quantification of efflux-encoding genes, (**a**) *acrB/ropD* in *K. pneumoniae* ATCC 51503 and (**b**) *mexA/ropD* in *P. aeruginosa* PAO1, untreated and treated with 0.5× MIC of esomeprazole (ESO), omeprazole (OME), and pantoprazole (PAN). Error bars explain the SD of three repeated runs, and the asterisks indicate highly significant difference with *** *p* < 0.0001 between untreated and PPIs-treated strains.

The data revealed remarkable downregulation of *acrB* expression in *K. pneumoniae* ATCC 51503 treated with 0.5× MIC of OME (91.5% reduction, *p* ˂ 0.0001), more than that challenged with 0.5× MICs of ESO and PAN (each 86% reduction, *p* ˂ 0.0001), compared with the untreated ATCC 51503 strain (Figure 8a). As illustrated in Figure 8b, *P. aeruginosa* PAO1 treated with 0.5× MIC of ESO, OME, and PAN showed brilliant down-expression in the *mexA* level by 95%, 99.7%, and 99.4%, respectively, compared with the PPI-free PAO1 strain (*p ˂* 0.0001).

### 2.6. Docking Analysis

**Figure 9:**
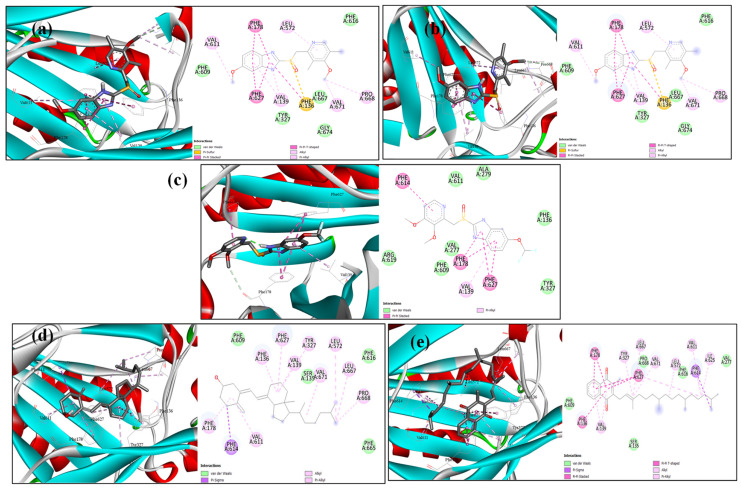
Docking of (**a**) esomeprazole, (**b**) omeprazole and (**c**) pantoprazole (**d**) vitamin D and (**e**) vitamin K into the active site of the AcrB receptor.

**Figure 10:**
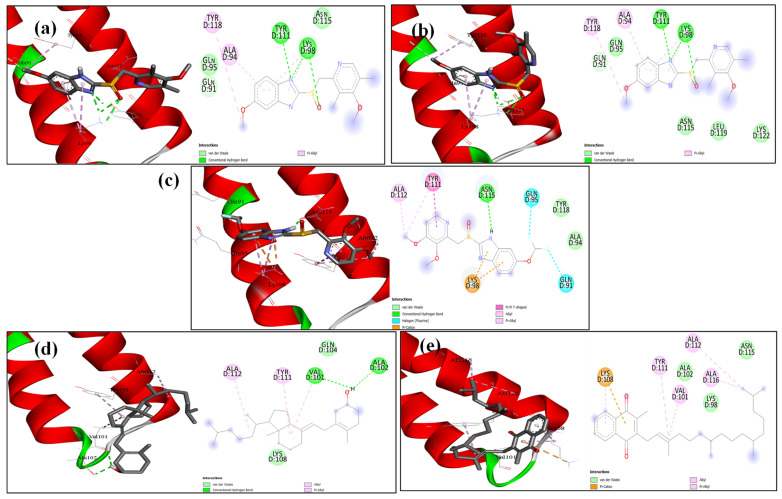
Docking of (**a**) esomeprazole, (**b**) omeprazole and (**c**) pantoprazole (**d**) vitamin D and (**e**) vitamin K into the active site of the MexA receptor.

The structures of AcrB and MexA proteins were subjected to molecular docking modelling to assess the potential mode of binding of the cocrystal ligand and the drugs omeprazole, pantoprazole, esomeprazole, vitamin D, and vitamin K. As anticipated, the docking score showed that every drug fits well into the active site region (Supplementary Table S1). The docking scores show that the five drugs’ binding was almost identical, varying between hydrogen bonding and solely hydrophobic interactions (Figure 9 and Figure 10). Remarkably, vitamin D has the greatest docking score in both the AcrB and MexA receptors (Figure 9 and Figure 10).

## 3. Discussion

Among Gram-negative bacteria, *K. pneumoniae* and *P. aeruginosa* are primarily opportunistic pathogens, causing a wide array of infections, and they can be zoonotic from animals and broiler chickens [29], nosocomial in hospitalized and immunocompromised individuals [30], and can cause community-obtained infections linked to the use of either spoiled food or impure water [31,32]. While in clinical settings, extensive work has been conducted on the risk associated with these organisms, they pose a significant threat to world health due to their success in the growing number of MDR phenotypes discovered each year that defy the currently available therapeutics [19].

The rise of MDR infections is partly attributed to the co-regulation of membrane impermeability and active drug efflux, which is believed to be a principal cause of both inherent and acquired drug resistance [8,33]. The e system is one of the serious determinants that most bacteria are armed with. Efflux pumps are membrane transporter proteins involved not only in pumping bacterial metabolites and harmful substances out to the external environment, but also in circumventing the action of several antimicrobial classes [34]. Extruding an antibiotic from the cells results in lowering its intracellular concentration, impairing its reaching its intended therapeutic target and causing treatment failure in addition to the great development of MDR phenomena [6,35,36].

The RND efflux pump is a major superfamily that has contributed to the overwhelming AMR with high priority in Gram-negative pathogens [37,38]. The main relevant efflux systems in *K. pneumoniae* and *P. aeruginosa* are AcrAB-TolC [39] and MexAB-OprM [40,41], respectively. Attenuation of efflux activity by EP-inhibiting agents is desirable since this will weaken the bacterial biofilm and virulence and successfully reconquer a vast swath of existing ineffective treatment options [42].

Although in recent research many EPIs have been identified, none of them can progress into clinical trials. Therefore, our research focused on studying the efficacy of already FDA-approved medications and supplements on the efflux system, hoping to repurpose their use as EPIs. According to the genotypic status, two standard strains of *K. pneumoniae* ATCC 51503 and *P. aeruginosa* PAO1 that were positive for *acrB* and *mexA* efflux genes, respectively (Figure 1), were utilized in this research.

Three clinically common proton uncouplers (ESO, OME, and PAN) and two vitamins (VD and VK) were examined for their MICs against the selected two standard strains using microtitre plate-based crystal violet assay [43]. Generally, the tested strains showed the same MICs for ESO, OME, and PAN, apart from MIC_ESO_ and MIC_PAN_ in PAO1 (Table 1). Overall, the antimicrobial activities of the three PPIs were at concentrations greater than their clinically desirable or achievable therapeutic doses. Administration of PPIs is associated with subsequent bacterial overgrowth, because they block gastric acidity secretions, which is necessary to hamper the reach of the infectious microbe to the gastrointestinal tract [44,45]. So, their inferior antibacterial performance (elevated MIC values) may be due to the development increase in bacterial non-susceptibility towards the PPIs. However, the slight antimicrobial effect of PPIs was likely attributed to their ability to inhibit the urease enzyme, which plays a vital role in bacterial colonization within the intestinal parietal cells, as well as their structural resemblance to some imidazole-based antibiotics (i.e., metronidazole and tinidazole) that work against bacteria [46]. Furthermore, the chelating capacity of PAN to Zn^+^ [2] ions could explain its inhibitory mechanism against metallo-β-lactamase producers [47]. Omeprazole, in a combination therapy, was more effective at killing MDR *H. pylori* in vivo and in vitro [48,49]. Some previous studies demonstrated the antibacterial activity of PPIs against *P. aeruginosa* [50], *M. tuberculosis* [51], and *S. aureus* [52], but further research is required to characterize the exact ability of PPIs by which they inhibit the bacterial growth in vitro.

Vitamins D and K showed more antimicrobial efficacy than PPIs, showing MIC ranges of 625 to 5000 (Table 1). Earlier studies have elucidated significant antibacterial effects of vitamins D and K, either alone or as a combination therapy, to fight resistant strains [53,54,55]. Vitamin D is an immune stimulator that improves the permeability of cell membranes and activates two small cationic peptide families, cathelicidin and defensin, that have broad-spectrum antimicrobial activities against a wide pathogenic range [56]. The bacteriostatic effect of VD on a wide variety of pathogenic Gram-positive and Gram-negative bacteria in addition to *C. albicans* was estimated [57,58]. Vitamin K has a great ability to alter the biological selective permeability function of the cell plasma membrane, so it limited the bacterial growth, and this signifies its antimicrobial and anti-efflux activity as well [28,59]. Vitamin K shows an antimicrobial activity against *S. aureus*, *S. agalactiae*, *S. pyogenes*, *B. anthracis*, *H. pylori* [60], and *P. aeruginosa* 03, exhibiting an MIC of 64 µg/mL, so potentiating the antimicrobial activity of aminoglycosides [59]. Not only do VD and VK have an anti-infective effect [61,62], but also vitamins B2 [63], C [64], P, and E [65].

We should take in account the side effects of PPIs, such as bone fractures, nutritional deficiencies, vertigo, backache, skin rash, and respiratory and gastrointestinal associated disorders [54,66]. The American Association of Poison Control Centers receives a startling 50,000 reports of vitamin toxicity each year. Other than hypervitaminosis-related signs such as tiredness, light-headedness, headaches, and digestive issues [67], the consequences are more pronounced for overdosing of VD due to soft-tissue calcification and brain, heart, and kidney damage [68] as well as of VK due to anaemia and haemorrhagic illness [22,69].

The efflux efficiency of the formerly chosen strains was supported by the fluorescent method, providing insights into the efflux of a substrate by the selected drug transporter-expressed strains and their sensibility towards the tested EPIs. Although EB emits trivial fluorescence, powerful strong fluorescence will emit when it enters and intercalates with the bacterial cell DNA. So, EB was selected as a broad range substrate for efflux investigation [70]. Based on MICs, all accumulation experiments were carried out with EB concentrations that did not exceed 0.5× MIC (Table 1) in order not to affect the cell viability, reproduction, or metabolic activity in any way.

Firstly, the steady-state EB concentration specific for each tested standard strain, *K. pneumoniae* ATCC 51503 and *P. aeruginosa* PAO1, was detected. This equilibrium concentration represented the balance between passive diffusion-associated EB entry and its extrusion by efflux pump systems. According to kinetic behaviour, activation of the bacterial efflux system begins to balance the passive diffusion-mediated EB influx. The amount of EB is extruded from the cytoplasm when it enters at low concentration that is not enough for irreversible binding with the cytoplasmic component. When the EB concentration surpasses its extrusion, it is embedded in nucleic acid within the cells, after which its extrusion is impossible [71]. Due to the overexpression of the efflux system in *K. pneumoniae* ATCC 51503 strain (AcrAB-TolC), it accumulated the least EB (1 μg/mL, Figure 2), even when it was exposed to the maximum 16 μg/mL of EB concentration. On the contrary, PAO1, whose MexAB-OprM efflux system has been inactivated, accumulated approximately 16 times more EB even when it was exposed to a far lower EB concentration (0.5 μg/mL, Figure 3).

We next demonstrated the activity of PPIs and vitamins on real-time EB accumulation in the previously chosen standard strains. All the employed drugs were tested at concentrations that do not inhibit the standard strains’ growth (sub-MICs) to ensure that any increase in EB fluorescence was not due to their antibacterial activity. In general, and as shown in Figure 2c and Figure 3c, the amount of EB accumulated by ATCC 51503 and PAO1, respectively, unexposed to glucose was higher than that treated with glucose (*p* ˂ 0.0001), indicating that glucose reenergizes the bacterial cells and maximizes active efflux. So, evaluating the EPI activities of the tested FDA-approved drugs and supplements (i.e., enhancement of EB accumulation) was performed under conditions that promoted the efflux machinery (presence of glucose and 40 °C incubation temperature). The potential efflux-inhibiting activity of the tested compounds was expressed through the calculated RFF, and the results were compared with values obtained for the reference CCCP control.

Upon addition of the tested PPIs and vitamins, the fluorescence level was increased, indicating an enhanced EB accumulation by the two reference strains compared with untreated standard cells, which was believed to be an important indicator for the speculated activity of the tested drugs [38]. The accumulation assays performed in the presence of the three PPIs showed that PAN was the most effective in retaining the efflux dye, more than the well-known CCCP in PAO1 (Figure 6c, Table 3) followed by ESO (Figure 6a, Table 3). On the other hand, in *K. pneumoniae* ATCC 51503, ESO (Figure 4a) accumulated the artificial EB substrate significantly more than OME, PAN, and even CCCP (Table 2).

The presence of 1/2 MICs of either VD or VK with ciprofloxacin significantly reduced the MIC of ciprofloxacin in both *P. aeruginosa* PAO1 and *K. pneumonia* ATCC 51503 by 64-fold. Furthermore, the combination of ciprofloxacin with PPEs showed a significant decrease in the MIC of ciprofloxacin, with a more than 32-fold decrease in the MIC of *P. aeruginosa* PAO1 isolate and a 4- to 32-fold decrease in MICs in ciprofloxacin/PPE combinations in *K. pneumonia* ATCC 51503.

There are different anti-efflux strategies, including (i) inhibition of substrate ejection either by competitive inhibition or by promoting some conformational changes, (ii) impairment of the outer membrane efflux component, and (iii) interruption of the functional assembly of the tripartite efflux protein structure [72]. The PPIs structure involves a substituted benzimidazole ring that irreversibly binds to and prevents the hydrogen–potassium ATPase enzyme, so affecting the bacterial drug uptake. Additionally, protonation of benzimidazoles in bacterial cytoplasm resulted in production of a reactive thiol group that binds with the efflux channel. Also, because of their proton blockage ability, they can inhibit the bacterial efflux using the proton (H^+^) gradient (drug/proton anti-porters) to extrude antibiotics to the cytosol. Hence, PPIs were considered potential EPIs by depleting the energy source required to eject the efflux substrate [3,73]. Our results agreed with the findings of Lake [74], who evaluated the anti-efflux activity of OME and PAN on rifampicin in *M. tuberculosis*. In line with previous reports, the anti-efflux efficiency of PAN was also inspected through reducing fluconazole resistance in *C. albicans* [75] or in all *Candida* spp. [76]. In congruence with many studies, the EP inhibition of OME was detected through fighting the microbial resistance and restoring the bactericidal activity of norfloxacin in *S. aureus* [52], attenuating *H. pylori* infection upon combination with the anti-emetic domperidone [77], increasing the antibiotic susceptibility in MDR *E. coli* by 2- to 8-fold, and the failure of EB to come out from more than 70% of bacterial isolates by the cartwheel method [78], in addition to decreasing the imipenem MIC up to 6-fold in *A. baumannii* [3]. In regard to virtual docking, OME was able to bind at a large hydrophobic bond, showing significant interactions with the MDR efflux system, NorA, in *S. aureus* [79]. Moreover, PPIs not only function as antacids, but they also block efflux pumps and enhance the effects of antibiotics by boosting their intracellular accumulation.

The fluorescence emission of cells incubated with glucose plus VD or VK showed a significantly higher degree of EB accumulated than that of cells incubated with glucose only, indicating inhibition of the efflux pump. Both VD and VK showed tremendous strengths of EB accumulation, particularly in PAO1 (Table 3 and Figure 7), but still less than the CCCP pump inhibitor. It was demonstrated that the association with VK significantly reduced the MIC of EB compared with EB alone, which was indicative of its efflux inhibition [28]. Both VD and VK are fat-soluble vitamins that induce morphophysiological changes in the cell membranes, resulting in loss of membrane potential or ions and, consequently, collapse of the proton pump with the depletion of ATP support. Additionally, VK, unlike other vitamins, fights the AMR by enhancing the cytoplasmic membrane stability and improving its permeability for antibacterial molecules in addition to hindering the bacterial efflux from pumping the antimicrobials out of the cells [28,59].

It is noteworthy that the effective EPI concentration of CCCP was 8000 µg/mL, those of the PPIs were 8000 and 16000 µg/mL, those of VD were 312.5 and 625 µg/mL, and those of VK were 1250 and 2500 µg/mL (Table 1). Therefore, VD and VK (which achieved the highest EPI activity with lower concentrations) were more potent than CCCP and the PPIs (which achieved their EPI activity with higher concentrations).

On the other hand, unlike CCCP, which has exhibited a toxicity problem by disrupting the mitochondrial proton-motive force in eukaryotes [80], the tested PPIs and vitamins are safer because they are extremely well tolerated and are consumed, all over the world, as OTC medications with very few adverse effects. Over and above that, screening and repurposing of FDA-approved PPIs and vitamins for having EPI action is a time-saving, inexpensive, and more auspicious approach than the strategy of de novo drug discovery that often has a high cost, unpredictable risk, and long development time.

The likelihood of bacterial resistance to EPIs must be taken into account due to mutations affecting the efflux system. EPIs were predicted to be substrates for bacterial pumps, so exposure to only the efflux inhibitor could be a way to put selective pressure on efflux inhibitor-resistant strains. As a consequence, the MIC of phenylalanyl-β-naphtylamide (PAβN) was increased by 6- to 12-fold in *K. pneumoniae* when combined with niclosamide due to a mutation in the *acrR* efflux gene [81].

On the other hand, using EPIs with significant antimicrobial effects is associated with serious bacterial resistance progression, so EPIs with no or little activity against the bacterial cell prolong the utilized drug’s lifespan via decreasing the resistance development [22,82]. For instance, the 1,8-naphthyridine is an EPI with no antibacterial activity, so bacteria are less likely to develop resistance towards it [82]. It is still important to confirm whether EPIs primarily target the efflux pumps rather than other side mechanisms, as this could inhibit or, at least, delay the bacterial resistance progression. PAβN, a broad-spectrum EPI, can increase the MIC of levofloxacin in *P. aeruginosa* by 8-fold, while in *P. aeruginosa*, it overexpresses MexAB-OprM transporters by 64-fold [83]. NSC 60339 compound intensifies erythromycin and novobiocin activity in *E. coli*, but it has no effect on these antibiotics in pump-lacking *E. coli* [84]. Even so, numerous EPIs have the ability to enhance antibiotic activity via efflux-independent mechanisms, such as impairing rather than penetrating the bacterial membrane. These EPIs not only can augment the antibiotic influx, but also be sufficient to induce bacterial lysis [85], resulting in the possibility for resistance development and cytotoxicity in clinical practice. Hence, for less development of EPI-mediated resistance, exploration of EPIs with no off-target effects, that penetrate the bacterial membrane rather than disrupt it, is crucial.

Owing to the inferior antibacterial efficacy of PPIs and their potency over the standard CCCP in the two selected reference strains, they were the most effective efflux inhibitors used. That is why qRT-PCR was performed, to deeply investigate the effect of ESO, OME, and PAN on bacterial efflux through quantifying the expression of the common efflux pump genes *acrB* and *mexA* in *K. pneumoniae* ATCC 51503 and *P. aeruginosa* PAO1, respectively. Although many studies have been carried out on the influence of many inhibitors on *acrB* and *mexA* efflux genes, unfortunately, no studies have evaluated the effect of PPIs on the function of these genes. Herein, the expression of the examined genes was significantly downregulated in PPI-treated strains compared with the untreated control. Although ESO, OME, and PAN were not less effective than each other, OME provoked a pronounced decrease in the expression level of *acrB* in *K. pneumoniae* ATCC 51503 and *mexA* in PAO1, by 91.5% (Figure 8a) and 99.7% (Figure 8b), respectively.

AcrAB-TolC is one of the well-characterized pumps contributing to fluoroquinolone-associated innate resistance in *K. pneumoniae*. The transporter AcrB is situated in the inner cytoplasmic membrane to function as the drug recognizer and selector in addition to energy transducer to facilitate the drug/proton anti-port process for the pump complex [86]. So, the loss of the *acrB* gene significantly reduced the colonization and ability of *Klebsiella* to cause pneumonia in mice [87]. It is one of the main virulence factors, so any decrease in *acrB* expression has a sturdy impact on lethal function, drug resistance and tolerance, and persistence of *K. pneumoniae* in the intestinal area [88].

In *P. aeruginosa*, MexAB-OprM is one of the largest outflow pumps, accounting for inherent multidrug resistance to anti-pseudomonal agents [89,90]. One of the structural components of this pump is the membrane fusion periplasmic protein MexA, which plays a crucial role in MDR phenomena due to the tight relation between elevated antibiotic non-susceptibility and *mexA* overexpression in *P. aeruginosa* clinical isolates [91,92,93]. Several previous research studies demonstrated that the reduction in *mexA* expression potentiates the activity of *P. aeruginosa* to ciprofloxacin [94], carbapenems [95], or towards a wide variety of antibiotic classes [19].

The downregulation in the relative gene expression of *acrB* and *mexA* may explain the efflux-abolishment effect of ESO, OME, and PAN. Altogether, the downturn in the expressive level of efflux-encoded genes reduced the number of efflux pumps on the bacterial cell membrane, resulting in shifting the antimicrobial drug from diminishment by efflux into enhancing its intracellular concentration. Even at low concentrations, it may exhibit potent bacteriostatic effects. Likewise, the efflux system represents a highly coveted target for combating bacterial-induced infections, while EPIs not only reduce bacterial resistance, but also decrease the incidence of development of resistant mutant strains.

These data were confirmed by docking analysis, which revealed high binding affinity of the tested vitamins and PPIs with both AcrB and MexA efflux regulatory proteins (Figure 9 and Figure 10). Similarly, both known efflux inhibitors PAβN and pyranopyridine bind efficiently to AcrB receptors via a hydrophobic trap [96]. In the study of Liu and coauthors, piperine eliminated efflux in *P. aeruginosa* through significant binding with the MexA receptor [95].

To date, no studies have been conducted on the role of ESO, OME, or PAN on the expression of efflux pump genes in Gram-negative bacteria or on identifying their putative binding efficacy to efflux pump components through computational docking techniques. Considering these factors, our study can herald using PPIs, VD, and VK, either alone or as antibiotic partners, to counteract the activity of bacterial efflux pumps and reduce the emergence of drug-resistant strains.

Therefore, this study provides prospective and promising potential for the usage of frequently consumed pharmaceutical supplements in the eradication of difficult-to-treat efflux-triggered infections. This study showed for the first time the potential anti-efflux actions of VD and VK, detected using representative *K. pneumoniae* and *P. aeruginosa* standard strains, with significant lowering of the MIC of ciprofloxacin. More verification and further in-depth research are needed to fully realize the potential of EPIs and to confirm these findings in other Gram-negative, Gram-positive, and even MDR organisms.

Additionally, due to in vitro investigation gaps, further experimental analysis is required in the quest to provide deeper future insights into the clinical applications of EPIs in vivo in light of their intricate mechanisms, off-target effects, cytotoxicity, and pharmacokinetics. Studying the compatibility of pharmacological characteristics and serum–drug half-life between EPIs and antibiotic partners is recommended too. More advanced techniques are also needed to test PPI- and vitamin-mediated efflux inhibition and determine the precise efflux machinery for each antibiotic to design suitable EPIs for these antibiotics. Also, clinical trials are crucial to rapidly re-position potential PPIs and vitamins as EPIs as an emergency response towards the antibiotic resistance calamity.

## 4. Materials and Methods

### 4.1. Bacterial Strains and Cultivation Media

Two laboratory standard Gram-negative strains, *K. pneumoniae* ATCC 51503 and *P. aeruginosa* PAO1, were used in this study. Luria–Bertani (LB) broth (10 g/L tryptone, 10 g/L NaCl, and 5 g/L yeast extract, pH = 7.2–7.4) is the most popular medium used for growth, multiplication, and preservation of these wild-type strains. Before each experiment, each strain was refreshed to restore mucoid lactose-fermenting pink colonies of *K. pneumoniae* ATCC 51503 on MacConkey’s agar and the distinct green colonies with appealing grape-like odour of PAO1 strain on cetrimide agar plates [97]. Both strains were stocked in LB with glycerol (50% *w*/*v*) in cryovials for long-term storage at −80 °C.

### 4.2. Chemicals and FDA-Approved Pharmaceutical Medications

Ethidium bromide (EB) and carbonyl cyanide m-chlorophenyl hydrazone (CCCP) were purchased from Sigma Aldrich (St. Louis, MI, USA). Three proton pump inhibitors (PPIs), esomeprazole (Chemipharm pharmaceuticals), omeprazole (Julphar pharmaceuticals), and pantoprazole (Sigma Pharmaceutical Industries, Al-Monofeya, Egypt).) were utilized as lyophilized powders (40 mg). Two vitamins, vitamin D (VD, 100,000 IU/mL), which was produced by Memphis Company, and vitamin K (VK, 10 mg/mL), the product of Amoun Pharmaceutical Co. S.A.E, were also tested.

### 4.3. Confirmation of Efflux Pump-Mediated Resistance

Two main efflux pump-specific genes for *K. pneumoniae* ATCC 51503 (*acrB*) and *P. aeruginosa* PAO1 (*mexA*) were investigated by uniplex PCR using the primer sequences and annealing temperatures listed in Table 5. Genomic DNA was rapidly extracted from both wild-type strains by boiling free cell suspensions at 95 °C for 10 min, followed by centrifugation, and the resulting cell-free supernatant was used as DNA template for PCR sets [98].

The amplification was performed in a standard 25 μL reaction mixture, and the negative control reaction, without bacterial DNA template, was also carried out. The cycling PCR conditions started with an initial denaturation of DNA at 95 °C for 5 min, followed by 35 cycles of denaturation at 95 °C for 30 sec, annealing for 30 sec, and extension at 72 °C for 1 min. The reaction was terminated by 1 cycle of final extension at 72 °C for 5 min. Positive efflux-correlated resistance was indicated by the appearance of clear, distinct DNA bands at the expected amplicon sizes, as detailed in Table 5, upon placing the agarose gel and capturing its image using UV gel documentation analytical system (Model Gel Documentation 1.4, 1189, AccuLab, New York, NY, USA).

### 4.4. Determination of the Minimum Inhibitory Concentrations (MICs) of the Tested Compounds

The minimum inhibitory concentrations (MICs), the lowest concentrations that showed no visible inoculum growth after 24 h incubation, of FDA-approved drugs and supplements, including three PPIs (ESO, OME, and PAN), two vitamin solutions (VD and VK), along with EB and CCCP, were assessed against the standard strains of *K. pneumoniae* ATCC 51503 and *P. aeruginosa* PAO1. The MIC determination was performed in flat-bottom microplate-based assay using micro broth dilution technique, and the results were interpreted regarding the CLSI breakpoints [43].

Aliquot (100 µL) of each PPI or vitamin was added in the first microplate well, and a series of 2-fold dilution increments was prepared in Mueller–Hinton broth (MHB) medium. About 0.01 mL of each diluted bacterial suspension was distributed in each well of the plate to give, approximately, a final density of 10^5^ CFU/mL. In each plate, MHB inoculated with untreated bacterial suspension and MHB only were prepared to serve as growth and sterility controls, respectively. The microtitre plates were incubated overnight at 37 °C for the final calculation of MICs and sub-MICs (0.5× and 0.25× MIC) for each drug that was used later in the efflux assays.

### 4.5. Quantitative Determination of Efflux Pump Inhibitory Effect of Some FDA-Approved Drugs and Supplements by Semi-Automated Fluorometric System

This assay was assigned to detect the real-time accumulation of an appropriate efflux pump substrate, ethidium bromide (EB), inside cells and its efflux using Rotor-Gene™ Q1000 thermocycler (Qiagen, Valencia, CA, USA) with the provided analytical real-time software, according to the procedure defined by Miguel Viveiros [103,104]. All tests were executed in triplicate.

### 4.6. Real-Time EB Accumulation Assay

An overnight growth of the two standard strains, *K. pneumoniae* ATCC 51503 and *P. aeruginosa* PAO1, were subcultured in 5 mL of nutrient broth medium and placed in a shaking incubator at 37 °C until they reached mid-log phase with an OD at 600 nm of 0.6. Cell pellets were collected from the broth by cooling centrifugation (4 °C) at 6000 rpm for 10 min, then washed twice and resuspended in phosphate-buffered saline (PBS, pH = 7.4) to obtain cellular suspensions with a final OD600 nm of 0.3 that matched 1 McFarland standard. Several PCR microtubes were prepared in a final volume of 100 µL, each containing the previously prepared bacterial suspension and 5 µL of varying EB concentrations ranging from 0.5 to 16 µg/mL with and without highly purified glucose solution (final concentration= 0.4% *w*/*v*). A microtube containing only EB in PBS at different concentrations was used as negative control in this assay.

To calculate the EB concentration that showed a detectable equilibration between its influx and efflux, all microtubes’ reaction mixtures proceeded at 40 °C in 36-well Rotor-Gene™ Q1000 thermocycler with the selection of appropriate excitation and emission wavelengths at 530 nm band-pass and 585 nm high-pass filters, respectively, to monitor the fluorescence every 1 min for 1 h. The increase in fluorescence over time was represented, and the steady state concentration of EB was calculated on a real-time basis, through dividing the relative florescence of (bacteria + EB + glucose) mixture by its cognate emitted by (EB + PBS) mixture [105].

### 4.7. Demonstration of the Activity of the Tested Compounds on Real-Time EB Accumulation

The calculated concentration of EB in the previous step, causing maximum accumulation, was further used to evaluate the activity of the tested FDA-approved drugs and supplements as EPIs. In 0.2 mL micro PCR tubes, aliquots of the formerly washed bacterial suspension (final OD600 nm = 0.3) supplemented with 0.4% *w*/*v* of highly purified glucose was added to 5 µL of EB (to give a final equilibrium concentration determined for each genus) along with the tested compounds (at their 0.5× MICs) and PBS to yield a final volume of 100 µL. Treated bacterial suspension with 0.5× MIC CCCP, a defined EPI, was prepared to serve as positive control tube. Another batch of untreated bacterial suspension (with no EPI) was carried out. A negative control reaction was also included as EB in PBS only.

All reaction microtubes were run in Rotor-Gene™ Q1000 thermocycler at 40 °C, and the fluorescence of EB was measured (excitation at λ530 nm and detection at λ585 nm) at the end of every cycle of 60 s, for 60 min total period. From the visualized real-time results, the efflux-inhibiting activity (successful retention of EB inside the cells) of each tested compound was described as the relative final fluorescence (RFF), which was calculated as follows: RFF = (RF_treated_ − RF_untreated_)/RF_untreated_, where RF_treated_ is the relative fluorescence (RF) of EB retention in bacterial suspensions treated with an inhibitor and RF_untreated_ is the relative fluorescence of EB retention in untreated control suspensions [106,107,108]. When the RFF value is above zero, this indicates maximum cell accumulation of EB in the presence of the tested compounds than those of the untreated control cells, and therefore, proves the inhibiting activity of the tested drugs and supplements on the bacterial efflux pump system [109,110].

### 4.8. Effect of Vitamins and PPIs on MIC of Ciprofloxacin

The MIC of ciprofloxacin against *K. pneumoniae* ATCC 51503 and *P. aeruginosa* PAO1 isolates was determined following the CLSI 2021 guidelines [43]. Ciprofloxacin was serially diluted by twofold, and MIC was detected alone and in combination with vitamins (VD and VK) and PPIs at 0.5× MIC. Each well was inoculated with 1 × 10^5^ CFU/mL of overnight cultures, and the plates were incubated overnight at 37 °C for detection of microbial growth.

### 4.9. RT-qPCR Assay

Quantitative RT-PCR was utilized to measure the effect of the tested PPIs, ESO, OME, and PAN, at their sub-MIC, on the expression level of efflux-based genes *acrB* and *mexA* in *K. pneumoniae* ATCC 51503 and *P. aeruginosa* PAO1, respectively.

The two reference strains, in the absence and presence of 0.5× MIC of each drug, were grown for 5–6 h with agitation at 150 rpm until the logarithmic phase of growth was reached. The cultures were then centrifuged at 4 °C for 20 min at 8000 rpm to collect the bacterial cells, and the total RNA was extracted using TRIzol reagent (Oxoid, Basingstoke, Hants, UK) based on the manufacturer’s instructions. Contaminating DNA was digested by RNase-free DNase I enzyme (Enzymonics, Co., Ltd., Daejeon, South Korea) according to the author’s recommendations, followed by precipitation of pure RNA with an equal volume of isopropanol and double washing with 75% HPLC–high-grade ethanol. The final RNA pellets were dissolved in 20 µL nuclease-free water (Sigma-Aldrich (Merck), Gillingham, UK). Next, the concentration and purity of RNA were estimated using the Nanodrop (BioDrop, Cambridge, UK), and cDNA was synthesized using a Thermo Scientific RevertAid First Strand cDNA Synthesis Kit (Thermo Scientific™, Loughborough, Leicestershire, UK).

RT-PCR analysis was performed using HERA Plus SYBR Green master mix and efflux gene-specific primers (Table 5). The relative quantity of mRNA corresponding to *acrB* and *mexA* genes was normalized to the reference rpoD genes in either *K. pneumoniae* and *P. aeruginosa*, respectively, and comparatively measured by the threshold cycle (CT) method [111] in a Rotor-Gene™ Q1000 thermocycler with real-time analytical software. Finally, fold change in each gene expression in treated cultures was compared with its expression in drug-free strain. Each isolate was repeated twice, and the calculated fold change values are the average of two independent runs.

### 4.10. Molecular Modelling Docking

The PDB was used to obtain the coordinates of the *K. pneumoniae* AcrB (PDB: 8FFS [112]) and *P. aeruginosa* MexA (PDB: 6TA5 [113]) efflux pump proteins. The three-dimensional structures of esomeprazole, omeprazole, pantoprazole, vitamin D, and vitamin K were drawn and optimized using Marvin Sketch [95] as part of the docking investigation. AutoDock Vina, version 1.1.2 (La Jolla, CA, USA) [114] was used to carry out docking operations. The active site’s coordinates (x, y, z) were 213.6/178.6/168.3 with size 20.6/20.4/20.7 for AcrB and 356.3/328.6/410.2 with size 30.0/24.3/33.1 for MexA. The 2D schematic presentation and 3D visualization were created using the Discovery Studio 2021 client [96].

## 5. Conclusions

In summary, this work focused on studying the most common Gram-negative bacilli in hospital settings: *K. pneumoniae* and *P. aeruginosa*. The significant increase in resistance, including MDR, reflects an emerging threat posed by these superbugs in our country. Our results revealed that the PPIs, in addition to the two vitamins D and K, exerted antimicrobial effects on the tested isolates at their pharmaceutical dosage. Moreover, they hindered the efflux machinery in the tested strains. The MIC of ciprofloxacin was also significantly reduced. This inhibitory effect was confirmed by the significant decrease in the expression of the genus-specific efflux-encoding genes *acrB* and *mexA*. This anti-efflux property of both drug categories holds significant value in clinical therapies against the tested strains. Co-administration of antibacterial agents with these FDA-approved drugs could reduce or reverse resistance. However, further research is required to elucidate the in vivo activity of these medications.

## Figures and Tables

**Table 1:** Minimal inhibitory concentrations (MICs) and sub-inhibitory concentrations (0.5× and 0.25× MICs) of the tested proton pump inhibitors and vitamins.

Category	Chemical/Pharmaceutical Medications	Bacterial Strains	MIC (µg/mL)	0.5× MIC (µg/mL)	0.25× MIC (µg/mL)
Reference efflux substrate	EB	*K. pneumoniae* ATCC 51503	2	1	0.5
		*P. aeruginosa* PAO1	32	16	8
Reference efflux inhibitor	CCCP	*K. pneumoniae* ATCC 51503	16,000	8000	4000
		*P. aeruginosa* PAO1	16,000	8000	4000
Proton pump inhibitors (PPIs)	ESO	*K. pneumoniae* ATCC 51503	16,000	8000	4000
		*P. aeruginosa* PAO1	32,000	16,000	8000
	OME	*K. pneumoniae* ATCC 51503	16,000	8000	4000
		*P. aeruginosa* PAO1	16,000	8000	4000
	PAN	*K. pneumoniae* ATCC 51503	16,000	8000	4000
		*P. aeruginosa* PAO1	32,000	16,000	8000
Vitamins	VD	*K. pneumoniae* ATCC 51503	625	312.5	156.25
		*P. aeruginosa* PAO1	1250	625	312.5
	VK	*K. pneumoniae* ATCC 51503	2500	1250	625
		*P. aeruginosa* PAO1	5000	2500	1250

EB: ethidium bromide, CCCP: carbonyl cyanide m-chlorophenyl hydrazone, ESO: esomeprazole, OME: omeprazole, PAN: pantoprazole, VD: vitamin D, VK: vitamin K.

**Table 2:** Relative final fluorescence (RFF) values of EB accumulation in *K. pneumoniae* ATCC 51503 standard strain in the presence of CCCP, PPIs, and vitamins.

C_Eq_= 1 µg/ml		
	**Compounds**	RFF ± SD
Standard EPI	CCCP	0.18 ± 0.09 ***
Proton pump inhibitors (PPIs)	Esomeprazole (ESO)	0.58 ± 0.06 ***
	Omeprazole (OME)	0.29 ± 0.13 ***
	Pantoprazole (PAN)	0.14 ± 0.14 ***
Vitamins	Vitamin D (VD)	0.092 ± 0.04 ***
	Vitamin K (VK)	0.06 ± 0.04 ***

C_Eq_: equilibrium concentration of EB, RFF: relative final fluorescence, SD: standard deviation. The results are the average of three independent experiments ± SD. Results were highly significant (*** *p* < 0.0001) compared with untreated cultures.

**Table 3:** Relative final fluorescence (RFF) values of EB accumulation in *P. aeruginosa* PAO1 standard strain in the presence of CCCP, PPIs, and vitamins.

C_E_q = 16 µg/mL		
	Compounds	RFF ± SD
Standard EPI	CCCP	15.6 ± 1.8 ***
Proton pump inhibitors (PPIs)	Esomeprazole (ESO)	21.03 ± 2.2 ***
	Omeprazole (OME)	19.70 ± 1.3 ***
	Pantoprazole (PAN)	29.9 ± 2.8 ***
Vitamins	Vitamin D (VD)	10.8 ± 1.4 ***
	Vitamin K (VK)	15.10 ± 1.3 ***

C_Eq_: equilibrium concentration of EB, RFF: relative final fluorescence, SD: standard deviation. The results are the average of three independent experiments ± SD. Results were highly significant (*** *p* < 0.0001) compared with untreated cultures.

**Table 4:** Minimal inhibitory concentrations (MICs) of ciprofloxacin (CIP) alone and in combination with the tested proton pump inhibitors and vitamins.

Category	Antibiotic/Utilized Medications and Supplements	Bacterial Strains	MIC (µg/mL)
Antibiotic alone	CIP alone	*K. pneumoniae* ATCC 51503	62.5
		*P. aeruginosa* PAO1	
Proton pump inhibitors (PPIs)	CIP/ESO	*K. pneumoniae* ATCC 51503	˂0.98
		*P. aeruginosa* PAO1	
	CIP/OME	*K. pneumoniae* ATCC 51503	15.6
		*P. aeruginosa* PAO1	˂0.98
	CIP/PAN	*K. pneumoniae* ATCC 51503	7.8
		*P. aeruginosa* PAO1	˂0.98
Vitamins	CIP/VD	*K. pneumoniae* ATCC 51503	3.9
		*P. aeruginosa* PAO1	0.98
	CIP/VK	*K. pneumoniae* ATCC 51503	7.8
		*P. aeruginosa* PAO1	1.95

CIP: ciprofloxacin, ESO: esomeprazole, OME: omeprazole, PAN: pantoprazole, VD: vitamin D, VK: vitamin K.

**Table 5:** List of primer sequences conducted in this study.

Organism	Gene	Primer	Sequence (5′→3′)	AT (°C)	Amplicon Size (bp)	Reference
*K. pneumoniae* ATCC 51503	*acrB*	AcrB F	CAATACGGAAGAGTTTGGCA	56	64	[99]
		AcrB R	CAGACGAACCTGGGAACC			
	*rpoD*	RpoD F	AAGACGAAGATGAAGACGCC	57	129	[100]
		RpoD R	CTTTGGCTTTGATGGTGTCG			
*P. aeruginosa* PAO1	*mexA*	MexA F	GTGACCCTGAATACCGAGC	60	144	[101]
		MexA R	GTCGATCTGGTAGAGCTGC			
	*ropD*	RopD F	CGAACTGCTTGCCGACTT	56	131	[102]
		RopD R	GCGAGAGCCTCAAGGATAC			

## Data Availability

All the data generated and analysed during the study are included in this manuscript. The raw datasets are available from the corresponding author upon request.

## Supplementary Materials

The following supporting information can be downloaded at: https://www.mdpi.com/article/10.3390/microorganisms13061227/s1, Table S1. Esomeprazole, Omeprazole, Pantoprazole, Vitamin D, and Vitamin K’s binding mechanisms and docking scores into AcrB and MexA receptors.

## Abbreviations

The following abbreviations are used in this manuscript:

*K. pneumoniae*	*Klebsiella pneumoniae*
*P. aeruginosa*	*Pseudomonas aeruginosa*
WHO	World Health Organization
MDR	Multidrug resistance
ABC	ATP-binding cassette
RND	Resistance–nodulation–cell division
SMR	Small multidrug resistance
MFS	Major facilitator superfamily
MATE	Multidrug and toxins extrusion
PACE	Proteo-bacterial antimicrobial complex efflux family
AMR	Antimicrobial resistance
EPIs	Efflux pump inhibitors
CCCP	Carbonyl cyanide m-chlorophenyl hydrazine
LB broth	Luria–Bertani broth
EB	Ethidium bromide
PPIs	Proton pump inhibitors
ESO	Esomeprazole
OME	Omeprazole
PAN	Pantoprazole
VD	Vitamin D
VK	Vitamin K
PCR	Polymerase chain reaction
MIC	Minimum inhibitory concentration
CLSI	Clinical Laboratory Standard Institute
MHB	Mueller–Hinton broth
PBS	Phosphate-buffered saline
RF	Relative final fluorescence
RT-qPCR	Real-time quantitative polymerase chain reaction
CT	Threshold cycle
ANOVA	One-way analysis of variance
