# Supplementary material for: Antimicrobial and Anti-Efflux Machinery of FDA-Approved Proton Pump Inhibitors and Vitamins Against Klebsiella pneumoniae and Pseudomonas aeruginosa
Source: Microorganisms. 2025 May 27;13(6):1227. doi: 10.3390/microorganisms13061227 (PMC12195029; doi:10.3390/microorganisms13061227)
Supplement: Supplementary file 1 [file microorganisms-13-01227-s001.zip › microorganisms-3529482-supplementary.pdf]

Antimicrobial and Anti-efflux Machinery of FDA-approved Proton Pump Inhibitors and Vitamins against *Klebsiella pneumoniae* and *Pseudomonas aeruginosa*

Lekaa L. Lutfi [1,2], Moataz A. Shaldam[3], Mona I. Shaaban[*1] and Soha Lotfy Elshaer[1]

1 Department of Microbiology and Immunology, Faculty of Pharmacy, Mansoura University, Mansoura 35516, Egypt

2 Department of Microbiology and Immunology, Faculty of Pharmacy, Horus University-Egypt, New Damietta, Egypt

3 Department of Pharmaceutical Chemistry, Faculty of Pharmacy, Kafrelsheikh University, Kafr El Sheikh 33516, Egypt

* Mona I. Shaaban

Mail; mona_ibrahem@mans.edu.eg

Supplementary table 1 ;

Table 1. Esomeprazole, Omeprazole, Pantoprazole, Vitamin D, and Vitamin K's binding mechanisms and docking scores into AcrB and MexA receptors.

Receptor	Compound	H-bonds	Hydrophobic interaction	Docking score
AcrB	Esomeprazole	--	Phe136, Val139, Phe178, Leu572, Val611, Phe627, Pro668, Val671	-7.8
	Omeprazole	--	Phe136, Val139, Phe178, Leu572, Val611, Phe627, Pro668, Val671	-7.9
	Pantoprazole	--	Val139, Phe178, Phe614, Phe627	-7.8
	Vitamin D		Phe136, Val139, Phe178, Tyr327, Leu572, Val611, Phe614, Phe627, Leu671, Pro668, Val671	-10.0
	Vitamin K		Phe136, Val139, Phe178, Tyr327, Leu572, Val611, Phe614, Phe627, Leu671, Val671, Ile625	-9.3
MexA	Esomeprazole	Lys98, Tyr111	Ala94, Lys98, Tyr118	-4.9
	Omeprazole	Lys98, Tyr111	Ala94, Lys98, Tyr118	-4.9
	Pantoprazole	Gln91, Gln95, Asn115	Lys98, Tyr111, Ala112	-5.1
	Vitamin D	Val101, Ala102	Val101, Tyr111, Ala112	-5.3
	Vitamin K	--	Val101, Lys108, Tyr111, Ala112, Ala116	-4.2
